# The Bovine Ex Vivo Retina: A Versatile Model for Retinal Neuroscience

**DOI:** 10.1167/iovs.64.11.29

**Published:** 2023-08-23

**Authors:** Jakub Kralik, Michiel van Wyk, Benjamin Leonardon, Giulia Schilardi, Sabine Schneider, Sonja Kleinlogel

**Affiliations:** 1 Institute of Physiology and Department for BioMedical Research (DBMR), University of Bern, Bern, Switzerland

**Keywords:** bovine retina, choroid-attached, ex vivo, research model, retinal ganglion cells

## Abstract

**Purpose:**

The isolated ex vivo retina is the standard model in retinal physiology and neuroscience. During isolation, the retina is peeled from the retinal pigment epithelium (RPE), which plays a key role in the visual cycle. Here we introduce the choroid-attached bovine retina as an in vivo–like model for retinal physiology. We find that—in the bovine eye—the choroid and retina can be peeled from the sclera as a single thin sheet. Importantly, the retina remains tightly associated with the RPE, which is sandwiched between the retina and the choroid. Furthermore, bovine tissue is readily available and cheap, and there are no ethical concerns related to the use of animals solely for research purposes.

**Methods:**

We combine multi-electrode array and single-cell patch-clamp recordings to characterize light responses in the choroid-attached bovine ex vivo retina.

**Results:**

We demonstrate robust and consistent light responses in choroid-attached preparations. Importantly, light responses adapt to different levels of background illumination and rapidly recover from photobleaching. The choroid-attached retina is also thin enough to permit targeted electrophysiological recording from individual retinal neurons using standard differential interference contrast microscopy. We also characterize light responses and membrane properties of bovine retinal ganglion cells and compare data obtained from bovine and murine retinas.

**Conclusions:**

The choroid-attached retinal model retains the advantages of the isolated retina but with an intact visual cycle and represents a useful tool to elucidate retinal physiology.

Millions of people worldwide suffer from retinal pathologies and blindness.^1^^–^^3^ Accordingly, pressure for new therapies is building.^4^^–^^6^ The best model for translational retinal research would be the human retina. Unfortunately, postmortem human retinas have a limited unforeseeable supply, underlie extensive ethical regulations, and require clinical collaboration to ensure tissue viability. Equally, human retinal organoids are not reliable research models. Despite recent advances, organoids lack intricate neural circuits—the basis of information processing in native retinal tissue—and have long maturation times.^7^^,^^8^ For this reason, the pre-clinical evaluation of new treatment strategies relies predominantly on mice.^9^^–^^11^ Adding to the load of this murine model, the ex vivo retina has become an immensely popular model in basic neuroscience and is now arguably the best understood part of the brain.^12^^–^^14^ Paradoxically, mice make a notoriously poor model for human vision: They are nocturnal and nonvisual animals with a visual acuity that lies far beyond the threshold for legal blindness in humans.^15^ On the other hand, wide availability and numerous disease models^16^ continuously advocate the use of mice.^17^

Perhaps the most fundamental shortcoming of the isolated ex vivo mouse retina is that the photoreceptor cells rapidly bleach to lose their sensitivity to light. This is caused by the disruption of the visual cycle (Supplementary Fig. S1A, S1B) between photoreceptors and retinal pigment epithelium (RPE), a thin single layer of epithelial cells that tile the outer edge of the retina.^18^ To ramify this, “dark-adapted” preparations are used: Research animals are kept in dark conditions before experiments, retinas are dissected either under dim red lighting or in the dark using special infrared-sensitive equipment, and light stimulation is always kept in the dim scotopic range to delay the bleaching process.^19^^,^^20^ Despite the power of this dark-adapted model,^13^ it has major drawbacks, which all stem from the lack of a natural visual cycle: (1) It is impossible to study natural light responses using photopic (daylight) light intensities or (2) how the retina natively adapts to different light-intensities. (3) The retina gradually loses light sensitivity. (4) There is a need for expensive specialized equipment, including night-vision goggles, microscopes with infrared optics, darkroom doors, and blinds.

Here we introduce the ex vivo bovine retina as a powerful and readily available model to investigate retinal physiology. Cattle are highly visual^21^^–^^23^ diurnal animals.^24^ The bovine retina has a visual streak adapted for relatively high-acuity vision.^25^ The bovine eye is comparable in size to the human eye (Supplementary Fig. S1C), with the ocular axial length being approximately 33 mm in cattle^26^ and 24 mm in humans.^27^ The murine ocular axial length in comparison is only 3 mm,^28^ which limits the amount of tissue available for experiments. Here, we were able to peel off the bovine retina with the choroid (Supplementary Fig. S1D). Choroid-attached explants had an intact visual cycle and an in vivo–like response to light.

Another attractive attribute of the ex vivo bovine retina is the fact that it is a waste product of the meat industry. This minimizes ethical concerns related to the use of animals solely for research purposes. Cattle meat production is currently higher than ever^29^ (Supplementary Fig. S1E), supporting the availability of the tissue. The bovine retina has been successfully used in studies that focus on protein purification^30^^–^^32^ and immunohistochemical assays^33^^–^^37^; however, apart from sparse electroretinogram (ERG) recordings,^38^^–^^40^ the bovine retina was entirely neglected as a model in retinal neuroscience (Supplementary Fig. S1E).

In this study, we establish and characterize light responses in the choroid-attached ex vivo retinal model using patch-clamp and multi-electrode arrays (MEAs). We show that light responses are resistant to photobleaching and adapt to ambient light intensities. We also characterize the alpha retinal ganglion cell types (RGCs) and report a discovery of a novel ON-OFF alpha RGC subtype in the bovine retina. This was followed by the quantitative comparison of murine and bovine light responses, as well as membrane properties at the level of RGCs. Taken together, our results explore a novel but at the same time accessible bovine model and advocate its use in retinal physiology and neuroscience.

## Methods

### Bovine Tissue and Preparation

**Figure 1.**
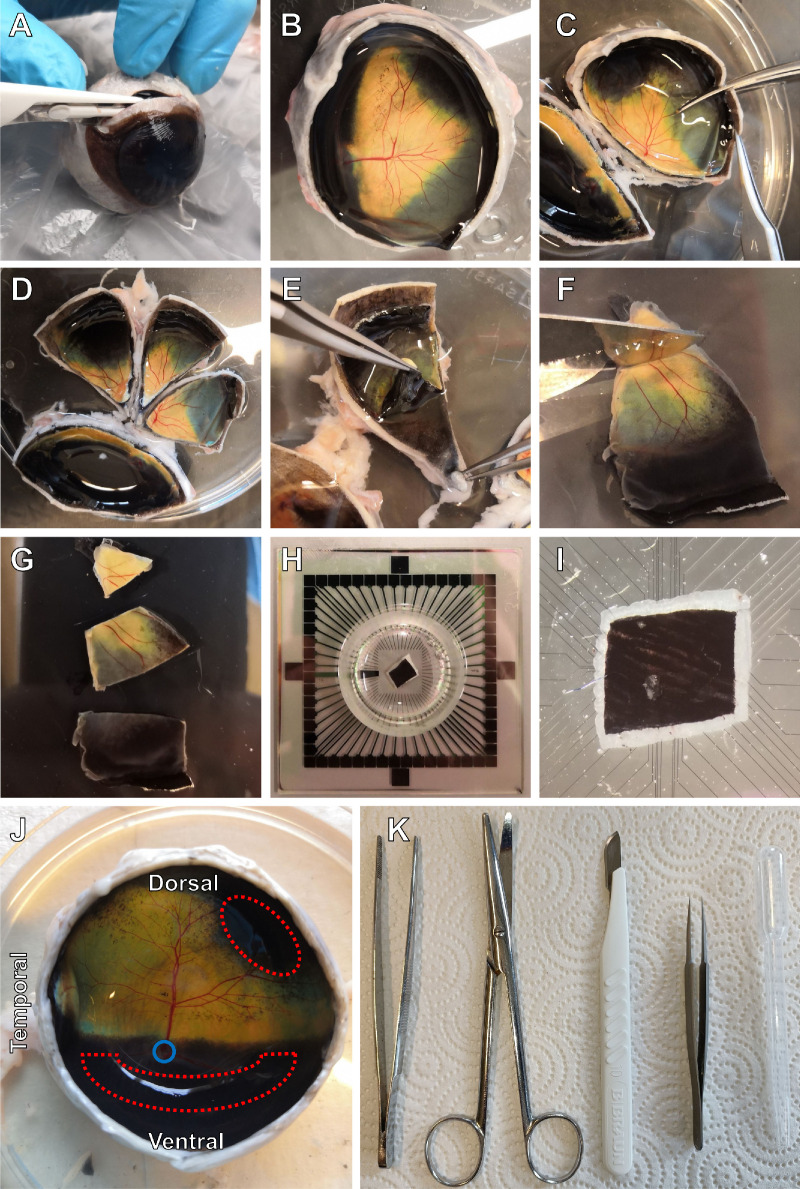
Procedure to obtain the choroid-attached ex vivo retinal explant. The bovine choroid with attached retina can be peeled from the sclera. (**A**) An incision is made ∼5 mm below the cornea of the freshly enucleated bovine eye to remove the lens and part of the vitreous before transportation to the laboratory. (**B**) The eye is moved to a fresh sucrose solution and opened using sharp dissecting laboratory scissors, preferably curved, but straight scissors work as well. The scissors are inserted into the previously created opening, and then the anterior part of the eye is removed, staying approximately 5 mm below the cornea. For this dissection procedure no magnification or dissecting microscope is needed. (**C**) Normally we use scissors to cut the eyecup into two parts for easier manipulation. In the next step the eyecup is cut into triangles, with the cut starting at the periphery. (**D**) Cutting this way ensures firm connection between the choroid and the retina. When we performed the cut from the center to the periphery, the choroid detached more often. (**E**) Choroid with attached retina is peeled from the eyecup, pulling from the center toward the periphery using fine forceps. (**F**) Choroid-attached retinal explant, vertical length approximately 4 cm. (**G**) Explants can be cut into smaller pieces using a scalpel blade. The cut must start from the edge, pressing first with the back of the blade. (**H**) Depiction of the choroid-attached retinal explant on the MEA, with the RGC layer facing the electrodes. (**I**) Detailed picture of choroid-attached retinal explant on MEA electrodes. (**J**) For electrophysiological recordings mid-periphery of the retina with strong RPE pigmentation was used (red dotted areas). *Blue circle* denotes the optic nerve. (**K**) Essential tools for the dissection of the bovine retina. From left: Dressing forceps, Dissection scissors, Scalpel blade no. 10, Dumont tweezers no. 5, Pasteur pipette with enlarged opening for tissue transportation. Illumination conditions of the dissection room were 300 lux.

Fresh bovine eyes were collected from a local abattoir (Metzg & Market Stefan Holzer, Hindelbank, Switzerland) three to eight minutes after euthanasia. Immediately on collection, eyes were hemisected along the corneal limbus to prepare eye cups that were transported to our laboratory (20 minutes) in a cold (on ice), oxygenated preservation solution^41^ containing (in mM) 200 sucrose, 21 NaHCO_3_, 10 glucose, 3 KCl, 1.25 NaH_2_PO_4_, 1.6 CaCl_2_, 2 MgCl_2_, 2 MgSO_4_ (pH adjusted to 7.4 pH). The dissection was performed in a fresh batch of the solution and completed in the scope of 10 to 15 minutes. The same cold, oxygenated preservation solution was then used to store choroid-attached preparations until electrophysiological recording. The time before the recording varied as one sample was used after another. We were able to reliably record electrical activity five hours after the dissection (light responses probably persisted for much longer periods, but this was not tested). The tissue used for electrophysiological recordings always originated from mid-periphery of the retina, specifically from dark-pigmented areas (Fig. 1J). For longer transportation periods, it is possible to use a mobile incubator.^42^

### Multi-Electrode Array Recordings

Retinas were placed RGC side down on MEAs (60MEA200/30iR-Ti; Multi Channel Systems MCS GmbH, Reutlingen, Germany) coated with Corning Cell-Tak Cell and Tissue Adhesive (Corning), with the ganglion cells facing the electrodes. The MEA recording device (MEA2100-System; Multi Channel Systems MCS GmbH) was coupled with a microscope (Zeiss Axioskop; Zeiss, Oberkochen, Germany). Tissue was continuously perfused with oxygenated, bicarbonate buffered Ames’ medium (36°C; 7 mL/min; Merck Millipore, Burlington, MA, USA), starting 30 minutes before the recording in darkness. The bottom illumination port of the microscope was equipped with a pE2 light stimulator (CoolLED Limited, Andover, UK) connected to a TTL signal generator (STG2008; Multi Channel Systems MCS GmbH) for the purpose of light stimulation, with intensity that is always denoted in the figure description. Recorded signals were collected, amplified, and digitized at 25 kHz using MCRack software (version 4.6.2; Multi Channel Systems MCS GmbH).

For the micro-ERG (mERG) analysis, the raw traces were filtered using a second-order Butterworth low-pass filter with a cutoff frequency of 20 Hz. Traces were extracted, down-sampled to 5000 Hz and analyzed offline using Matlab (version R2021b; MathWorks, Inc., Natick, MA, USA). In our analysis, we focused on the maximal negative deflection of the A-wave. This was done by targeting the first 50 ms after the onset of light stimulation. To calculate the amplitude of the A-wave, we subtracted the average basal activity (recorded one second before the light stimulation) from the maximal negative deflection occurring in the first 50 ms of light stimulation. Each electrode was treated as an individual unit. Statistical analysis was performed in GraphPad (Prism, version 9.3.1; GraphPad, San Diego, CA, USA) using the Friedman test with multiple comparisons.

Extraction of single-cell light responses at the level of RGCs was done as described elsewhere.^43^^,^^44^ In brief, the raw traces were filtered using a second-order Butterworth high-pass filter with a cutoff frequency of 200 Hz and extracted spikes were spike-sorted (Offline Sorter, version 4.6.0; Plexon, Dallas, TX, USA).

The description of analysis procedures is included in the Supplementary Material and Methods. Statistical analysis was performed in GraphPad (Prism, version 9.3.1) using the Mann-Whitney test with multiple comparisons. Normality was rejected for all datasets via both Shapiro-Wilk test and Kolmogorov-Smirnov test.

### Single-Cell Recordings

Choroid-attached retinas were placed RGC side up inside the recording chamber. Tissue was perfused with gassed, bicarbonate buffered Ames’ medium (36°C; 7 mL/min; Merck), starting 30 minutes before recording, and stabilized with a stainless-steel harp with Lycra threads (model no. HD-42/15; Warner Instruments LLC, Hamden, CT, USA). Electrodes were pulled from borosilicate glass to a final resistance of 5 to 8 MΩ. For the recordings of light responses from bovine alpha RGCs, cell-attached and whole-cell patch-clamp recordings were performed under direct visual control using a Carl Zeiss Axio Examiner 1D microscope fitted with commercial far-red differential interference contrast (DIC) optics (750–790 nm) and an Axiocam 702 mono camera.^44^^,^^45^ The glass pipettes were filled with Ames’ medium. The recordings were made using a HEKA EPC10 amplifier with PatchMaster software. Light stimuli were generated by a pE-4000 system (CoolLED Limited) and projected through a 20× water immersion objective onto the retina. The stimulus period was triggered directly by the PatchMaster software. Stimulus intensity was controlled using the pE-4000 system and neutral density filters in the light path.

For the electrophysiological comparison of active and passive membrane properties of murine and bovine alpha RGCs, whole-cell current-clamp recordings were performed using a Nikon eclipse E600FN microscope equipped with a 40× water immersion objective (Fluor TM; Nikon Inc., Melville, NY, USA) connected to an Infrared Altairastro camera. Pipettes were filled with an intracellular solution containing (in mM) 115 K-gluconate, 5 KCl, 5 EGTA, 10 HEPES, 2 Na-ATP, and 0.25 Na-GTP. Recordings were performed with an Axopatch 200B amplifier (Molecular Devices, San Jose, CA, USA). Recordings were low-pass filtered (5 kHz) and acquired with Clampex software (Molecular Devices, San Jose, CA, USA). Current traces were digitized (10 kHz) and stored on the hard drive of a personal computer. The data collected were analyzed offline with clamp fit 10.4 (Molecular Devices). In total, nine parameters of passive and active membrane properties were compared (Table) as described in detail in the supplementary materials file.

**Table.** Individual Data of Mice and Bovine RGCS Intrinsic Properties

	Vm (mV)	Rn (MΩ)	T (ms)	Sag (mV)	Steady Freq (Hz)	Max Freq (Hz)	FA	Amplitude (pA)	SW (ms)
Bovine RGCs									
GC 1	−53	130	14.3	−8.78	27.9	149.3	0.81	107.8	0.86
GC 2	−52	81	4.1	−2.97	54.6	166.7	0.67	91.4	0.80
GC 3	−56	159	11.7	1.13	35.6	111.0	0.68	76.1	1.15
GC 4	−58	324	17.4	−1.08	22.4	65.4	0.66	59.0	1.43
GC 5	−55	49	44.1	1.75	43.2	80.9	0.47	47.7	1.01
GC 6	−60	158	41.5	−5.24	18.2	52.7	0.65	89.5	1.17
GC 7	−57	95	53.2	−7.99	51.4	176.5	0.71	103.5	0.54
GC 8	−60	79	41.7	−7.44	59.0	168.7	0.65	103.3	0.57
GC 9	−59	207	81.6	−6.53	49.9	114.5	0.56	89.6	0.99
GC 10	−55	133	42.4	−7.24	45.5	130.3	0.65	90.4	0.72
Mean	−56.5 ± 0.9	141 ± 25	35.2 ± 7.4	−4.44 ± 1.2	40.8 ± 4.5	121.6 ± 14.1	0.65 ± 0.03	85.8 ± 6.2	0.92 ± 0.1
Mouse RGCs
GC 1	−54	700	80.3	−12.19	30.2	75.7	0.60	61.0	2.02
GC 2	−53	496	28.8	−6.30	26.5	80.8	0.67	97.8	1.37
GC 3	−51	244	19.1	−6.98	48.6	135.5	0.64	78.2	0.94
GC 4	−46	113	60.0	−3.71	52.4	137.0	0.62	87.4	1.08
GC 5	−50	526	24.2	−4.67	25.8	80.0	0.68	68.3	1.56
GC 6	−47	499	179.3	−7.33	58.8	110.0	0.47	46.2	1.18
GC 7	−57	146	7.5	−4.43	107.2	210.4	0.49	86.6	0.94
GC 8	−60	758	29.4	−6.18	41.7	98.4	0.58	63.6	1.05
GC 9	−54	711	44.8	−7.59	45.1	98.9	0.54	82.4	1.66
Mean	−52.4 ± 1.5	465 ± 82	52.6 ± 17.5	−6.60 ± 0.8	48.5 ± 8.3	114.1 ± 14.2	0.59 ± 0.02	74.6 ± 5.4	1.31 ± 0.1

*RGCs,* Retinal ganglion cells*; V*_m_, resting membrane potential; *R_n_*, input resistance; *τ,* time constant; *Sag,* rectification of *V*_m_ back toward resting level in response to hyperpolarization; *Steady Freq*, Steady firing rate frequency*; Max freq,* Maximal firing rate frequency; *FA*, frequency adaptation index; *Amplitude*, spike amplitude; *SW*, spike width.

Statistical analysis was performed with GraphPad (Prism, version 9.3.1) using unpaired *t*-testing. Data are presented as means ± standard error to the mean.

## Results

### In Vivo–Like Retinal Physiology in the Choroid-Attached Ex Vivo Retina

We first established a dissection protocol where the choroid is peeled from the bovine eye cup without any retinal detachment (Fig. 1). Once this was achieved, we studied light adaptation in choroid-attached retinal explants, using both MEA and single-cell patch-clamp recordings.

**Figure 2.**
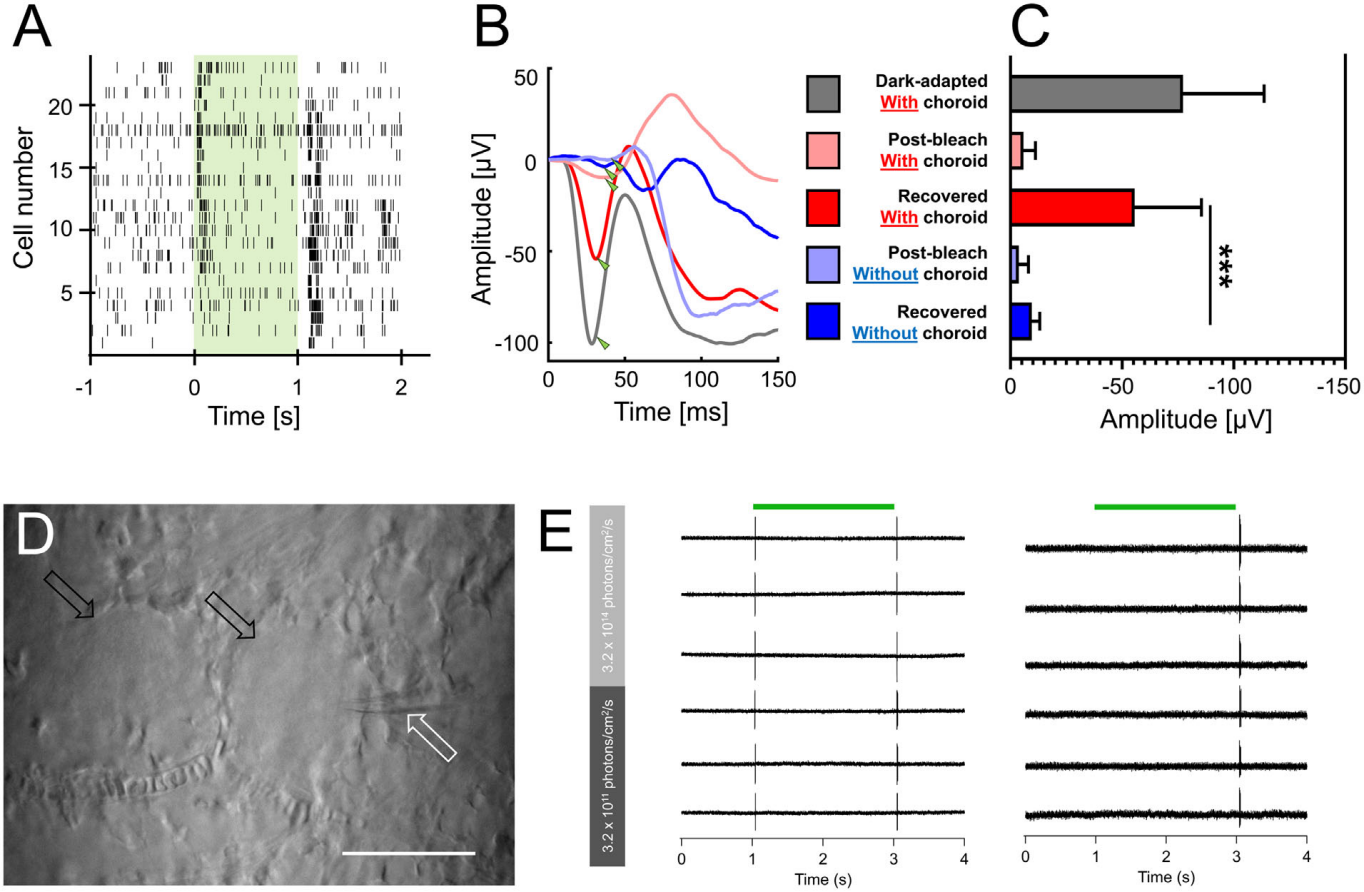
MEA and patch-clamp recordings from the choroid-attached ex vivo retina. (**A**) Example RGC-derived raster plots (n = 23 cells) in response to light stimulation (550 nm; one second; 3.2 × 10^15^ photons/cm^2^/s). (**B**) Representative mERGs in response to full-field light flashes (550nm; one second; 3.2 × 10^15^ photons/cm^2^/s) at different stages of the light stimulation protocol. *Green triangle* indicates the position of the A-wave. The A-wave is practically absent immediately after photobleaching; however, it recovers in the choroid-attached ex vivo retina. (**C**) Comparison of the A-wave amplitude at different stages of the light-stimulation paradigm derived from nine retinal explants (n = 401 electrodes). Immediately after photobleaching the amplitudes of A-waves did not differ significantly (with choroid = −5.7 ± 5.43 µV; without choroid = −3.7 ± 4.33 µV; *P* = 0.13). The A-wave recovers on average to more than 70% (−55.6 ± 29.93 µV) of its original, dark-adapted, values (−77.2 ± 36.3 µV) in five minutes in a choroid-attached ex vivo retinal explant, whereas it only reaches 12% (−9.5 ± 3.63 µV) of the original amplitude at the same time span in choroid-devoid explant. Comparison of the recovered amplitudes with and without RPE resulted in a significant decrease without the RPE (*P* < 0.001) (**D**) Individual ganglion cells (*black arrows*) and fine structures (e.g., capillaries with erythrocytes) are clearly visible through far-red DIC optics. A glass recording pipette (*white arrow*) is positioned against one of the RGCs for recording. *Scale bar:* 100 µm. (**E**) Cell-attached light responses from individual RGCs targeted for recording using direct visual control. For each cell, recordings were made at two different background light intensities.

In MEA recordings, bovine RGCs had robust spike responses to a step change in light intensity (Fig. 2A). However, for a direct readout of the photoreceptor response—and indirect readout of unbleached opsin—we extracted the mERG^43^^,^^46^ frequency band and focused specifically on the A-wave, which encodes the light-evoked hyperpolarization of the photoreceptor cells.^47^

A test step in light intensity triggered a robust mERG in the dark-adapted retina (Fig. 2B). When the retina was subsequently photobleached (Supplementary Fig. S2) with high-intensity light, the mERG triggered by the same test step, 15 seconds after bleaching, were devoid of an A-wave. Importantly, after only a five-minute period of dark recovery, the mERG A-wave reappeared. Dark recovery of the photoreceptor response infers an intact visual cycle. Next, we carefully peeled the choroid/RPE from the explant using fine forceps and reiterated the same photobleaching experiment (Supplementary Fig. S2). As anticipated, without the RPE, the visual cycle was disrupted, and A-wave recovery was compromised after photobleaching.

Statistical analysis (Fig. 2C; n = 401 electrodes from nine retinas) shows that the A-wave amplitude was reduced after photobleaching, with no significant difference between choroid-attached and isolated retinas (15 seconds after photobleaching; with choroid = −5.7 ± 5.43 µV; without choroid = −3.7 ± 4.33 µV; *P* = 0.13). Small persisting currents most likely stem from cone photoreceptors, which are much harder to bleach than rod photoreceptors. In choroid-attached preparations, the A-wave amplitudes increased ∼11-fold (−55.6 ± 29.93 µV) from those observed immediately after photobleaching. Once the RPE was removed, the A-wave reached only ∼threefold increase (−9.5 ± 3.63 µV) of its original photobleached amplitude. The difference in bleach recovery between choroid-attached and isolated retinas, as expected, was highly significant (*P* < 0.001; Friedman's paired multiple comparison test). Results of all statistical tests can be found in Supplementary Table S1. Additionally, we also compared the ability of intact preparations (with RPE) and isolated preparations (without RPE) to recover after the photobleaching period (Supplementary Fig. S3). We observed almost 70% recovery of the A-wave component of mERGs in the intact preparations, whereas the recovered amplitudes in isolated preparations reached only 9% of the original amplitude.

Next, we wanted to test whether choroid-attached explants can be used in targeted single-cell electrophysiology. Despite strong pigmentation of the RPE and choroid, individual cells were clearly visible and were easily targeted under visual control (Fig. 2D) using far-red DIC optics (770 nm). To demonstrate efficient light intensity adaptation, we recorded cell-attached responses from RGCs to step changes in light intensity at two levels of background illumination (Fig. 2E; 3.2 × 10^14^ and 3.2 × 10^11^ photons/cm^2^/s; stimulus contrast defined as C = (L_max_ − L_min_)/(L_max_ + L_min_) was set between 0.8 and 0.9). Recordings were first made at the bright background before subsequent adaptation (10 minutes) and recording at the low background intensity. RGC light responses remained conserved at different background luminosities, suggesting effective adaptation to ambient light and supporting the hypothesis of an intact visual cycle.

### A Novel Alpha Cell Type in the Bovine Retina

**Figure 3.**
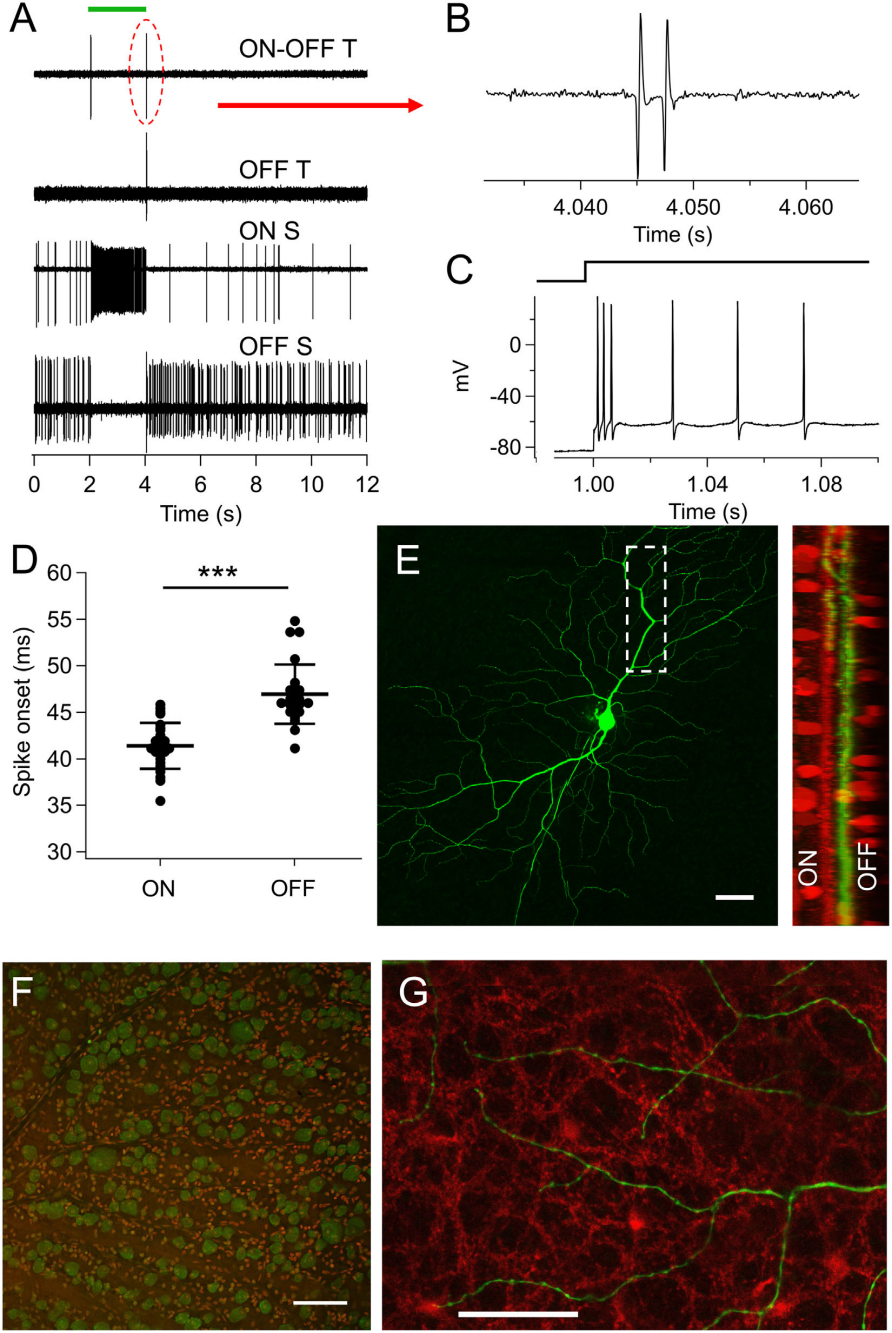
A novel Alpha cell type in the bovine retina. (**A**) Representative traces of major alpha cell types in the bovine retina. The new ON-OFF transient (ON-OFF T) alpha cell is not found in mouse retina (*top*). (**B**) ON-OFF T cells always fire action potential bursts with two or three spikes. (**C**) Current injection in patch-clamp experiments demonstrated that spikes are equally exhausted after an initial burst of two to three spikes. (**D**) The spike onset of ON-OFF T cells is significantly faster at the onset of a light stimulus compared to the end (*P* < 0.001). (**E**) Intracellular dye labeling of ON-OFF T cell. The size of the dendritic-field diameter is over 1 mm with stratifications predominantly in the OFF sublamina, with some dendrites extending to the ON sublamina of the inner plexiform layer (*right*). *Scale bar:* 100 µm. (**F**) Labeling against RBPMS (*green*) shows clear distinction of RGCs. Nuclear stain in *red*, *scale bar:* 100 µm. (**G**) Dendrites of the ON-OFF alpha cell (*green*) do not co-fasciculate with the plexus of cholinergic amacrine cell dendrites (*red*). *Scale bar:* 50 µm.

When targeting RGCs with large cell-bodies, we find four Alpha-type RGCs in the bovine retina (Fig. 3A). These include the ON-sustained, OFF-sustained, and OFF-transient types—described previously in the murine retina^19^^,^^48^—as well as a large ON-OFF transient (ON-OFF T) cell type. ON-OFF T cells always fire action potential bursts with two or three spikes on light stimulation (Fig. 3B). Injecting current shows that spikes are equally exhausted after an initial burst of two to three spikes (Fig. 3C), inferring that the spiking pattern of ON-OFF T cells is largely an intrinsic postsynaptic property. The spike onset of ON-OFF T cells is fast, with a faster onset of spiking at the onset of a light stimulus compared to the end (Fig. 3D; *P* < 0.001).

Intracellular dye labeling shows that the ON-OFF T cells are large with dendritic-field diameters over 1 mm. Co-labeling with choline acetyltransferase showed dendritic stratification predominantly in the OFF sublamina, with some peripheral dendrites extending to the ON sublamina of the inner plexiform layer (Fig. 3E). In addition to choline acetyltransferase, known immunomarkers for retinal cell types worked well in the bovine retina (Supplementary Fig. S4; Supplementary Table S2). RGCs were easily distinguishable from displaced amacrine cells (Fig. 3F). The dendrites of the ON-OFF alpha cells do not co-fasciculate with the plexus of cholinergic amacrine cell dendrites (Fig. 3G).

### Comparison of Murine and Bovine Light Responses and Electrophysiological Properties

**Figure 4.**
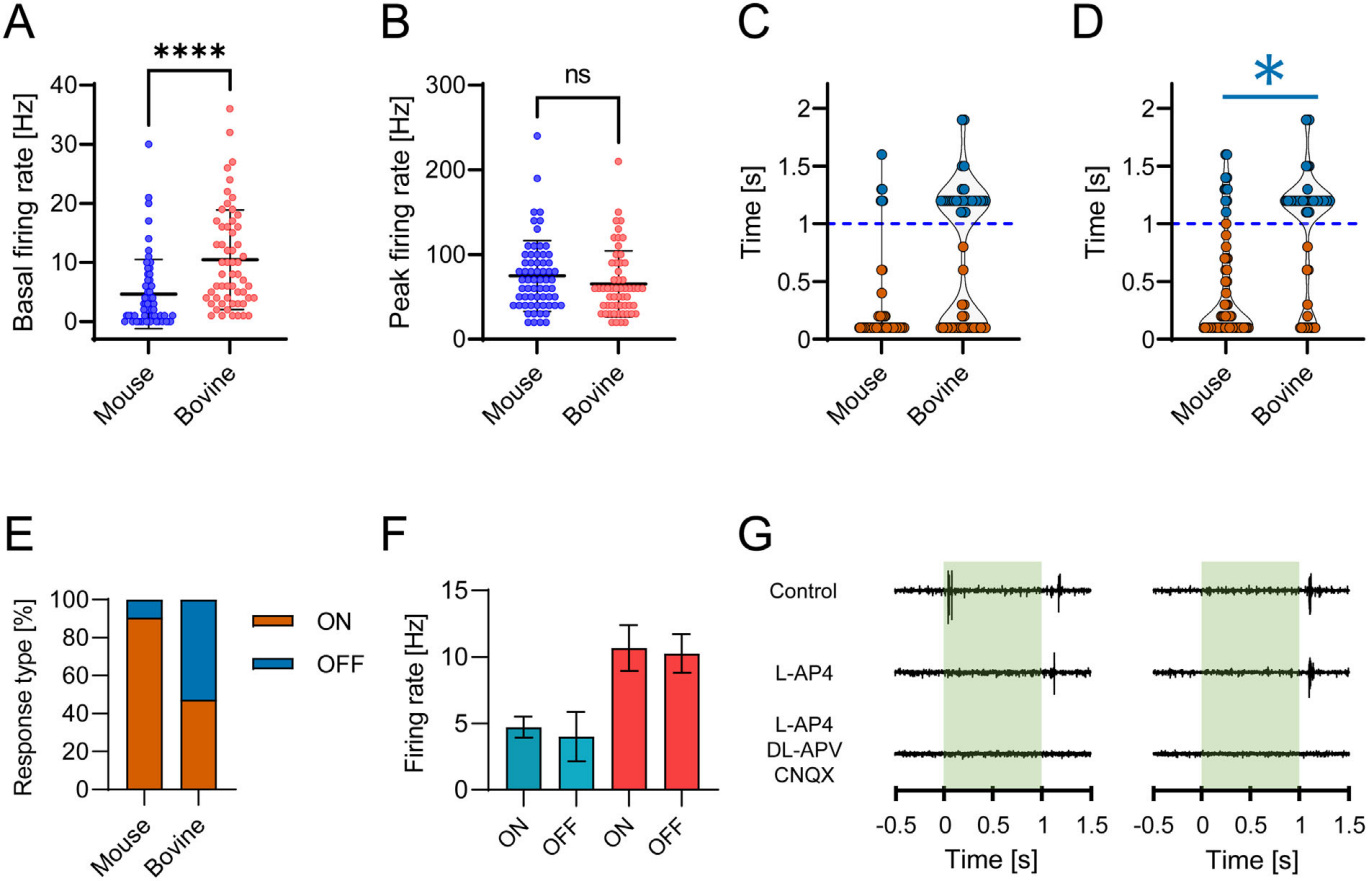
Functional comparison of murine and bovine RGCs using MEA recordings. (**A**) Comparison of basal firing rate of mouse (n = 64 cells; 4.64 ± 5.86 Hz) and bovine RGCs (n = 57 cells; 10.46 ± 8.41 Hz) shows significant differences (****P < 0.0001). (**B**) The same RGCs, however, did not show differences in their peak firing rates upon light stimulation (*P* = 0.1329; mouse = 74.69 ± 41.71 Hz; bovine = 65.44 ± 39.10 Hz). Points in plots correspond to individual cells, thicker *black horizontal bar* represents mean ± SD. Comparison of onset (**C**) and time-to-peak (**D**) of the light responses between mouse (n = 64 cells) and bovine (n = 57 cells) light-responsive RGCs. We did not observe significant differences between RGCs with onsets of light responses (**C**) during light stimulation (*orange points*; *P* = 0.58; mouse = 136 ± 102 ms, n = 54; bovine = 167 ± 166 ms, n = 16) nor after it (blue points; *P* = 0.19; mouse = 300 ± 155 ms, n = 6; bovine = 263 ± 194 ms, n = 30). The responses that peaked during light stimulation (**D**) did not show any significant temporal time differences (*orange points*; *P* = 0.61; mouse = 243 ± 239 ms, n = 58; bovine = 225 ± 230 ms, n = 27). However, responses that peaked after the light stimulation exhibited faster kinetics in bovine retinas in comparison to mouse retinas (*blue points*; *P = 0.022; mouse = 340 ± 165 ms, n = 10; bovine = 268 ± 199 ms, n = 41). *Blue dashed horizontal line* represents the offset of light stimulation (starting at time 0). (**E**) Bar plot showing the relative fraction of receptive-field types recorded in the mouse (n = 64 cells) and bovine retina (n = 57 cells). Compared to the mouse retina, we observed shift from ON to OFF response types. (**F**) Comparison between the basal firing rates ON and OFF responses (Mouse: ON = 4.71 ± 6.01 Hz, OFF = 4.00 ± 4.56 Hz 6 cells; Bovine: ON = 10.67 ± 9.04 Hz, OFF = 10.27 ± 7.96 Hz) in each species did not yield significant differences (Mouse: ON = 58 cells, OFF = 6 cells, p = 0.914; Bovine: ON = 27 cells, OFF = 30 cells, *P* = 0.997). (**G**) Exemplar raw MEA traces showing ON-OFF (left) and OFF (right) light responses from the bovine retina. Application of L-AP4 abolishes the ON component, whereas the OFF component remains. Additional application of DL-APV and CNQX effectively blocked the OFF component as well. For all shown statistical analysis Mann-Whitney testing was used, preceded by rejection of normality via both Shapiro-Wilk and Kolmogorov-Smirnov tests.

**Figure 5.**
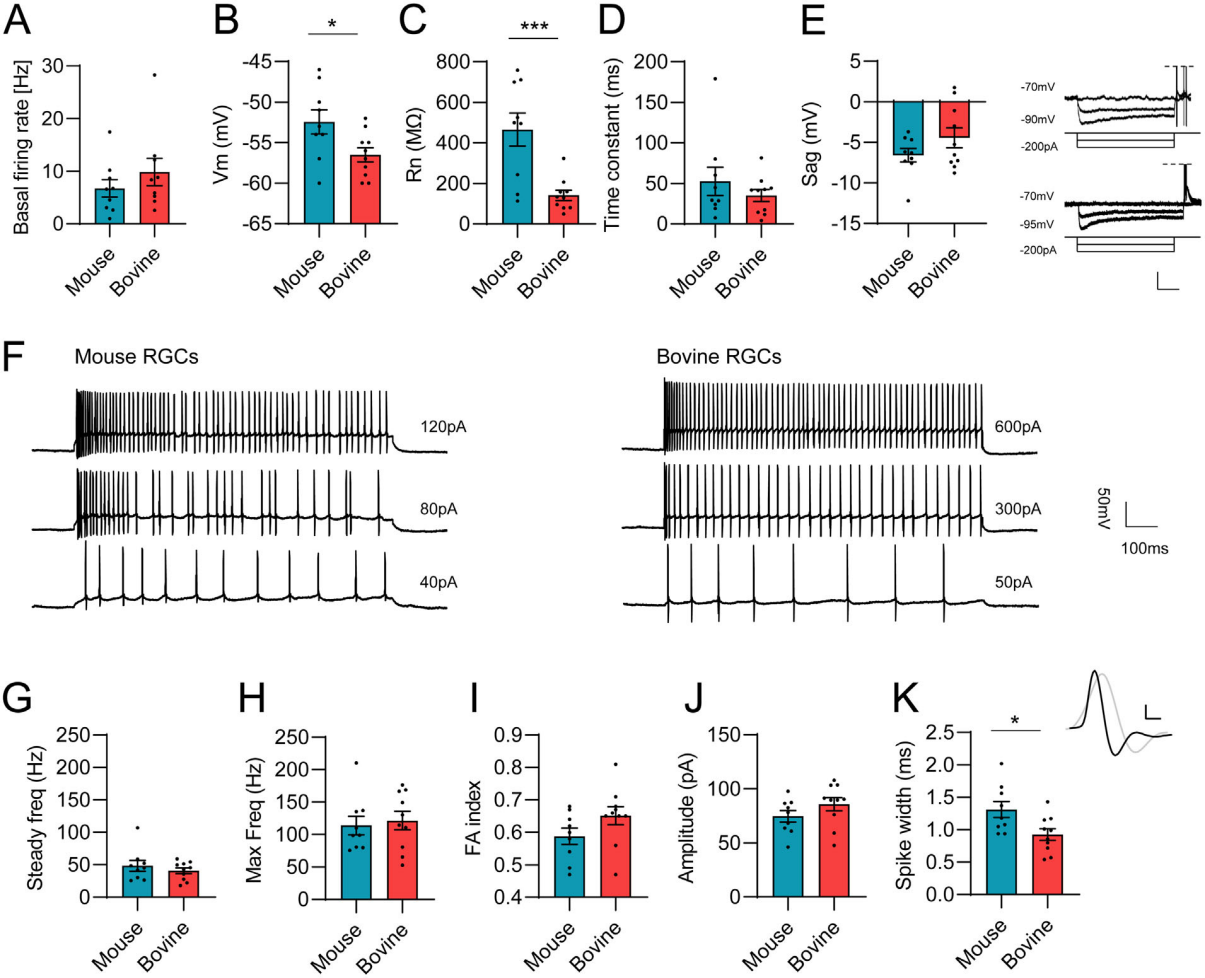
Intrinsic physiological properties of murine and bovine RGCs. Patch-clamp analysis of (**A**) basal firing rate, (**B**) membrane potential, (**C**) input resistance, (**D**) time constant, (**E**) anomalous rectification in response to hyperpolarization “sag”, (**G**) steady firing rate frequency, (**H**) maximum firing rate frequency, (**I**) frequency adaptation index, (**J**) spike amplitude and (**K**) spike half-width. (**E**) Right: Representative traces of bovine (top) and mice (bottom) RGCs in response to hyperpolarizing current injections. At the end of hyperpolarizing current, most cells exhibited rebound bursting (top right). *Vertical scale bar*, 20 mV; *horizontal scale bar*, 200ms. (**K**) *Top*: Average bovine (*black*) and mice (*gray*) action potential (from 100 spikes) in response to depolarizing current injections. Vertical scale bar, 20 mV; horizontal scale bar, 1 ms. (**I**) Representative spiking patterns of mice (*left*) and bovine (*right*) RGCs in response to depolarizing current injected for one second at threshold level (*bottom*), midrange (*middle*), and maximum firing rate (*top*). All RGCs recorded from bovine, or mice exhibit a regular spiking pattern with a decrease of firing rate over time. *Vertical scale bar:* 50 mV; *horizontal scale bar*: 100 ms. Individual and averaged values summarized in the Table.

In this set of experiments, we wanted to compare the overall RGC output of the bovine retina with that of mice using MEA recordings. For a more accurate comparison, we peeled away the choroid from the bovine retina after dark adaptation (30 minutes) in the recording setup for MEA recordings. For murine retinas all the procedures of dissection were done in the dark (see Supplementary Material and Methods). We first compared the basal firing rates of both murine and bovine light-responsive RGCs before any light stimulation (Fig. 4A). Murine RGCs exhibited spontaneous activity with frequency ∼5Hz (4.64 ± 5.86 Hz; n = 5 retinal explants; n = 64 cells), as previously reported.^43^^,^^49^^,^^50^ We observed significantly higher (*P* < 0.0001) basal activity of the bovine RGCs (10.46 ± 8.41 Hz; n = 4 retinal explants; n = 57 cells). Similar observations, albeit not significant, were observed in the single-cell patch-clamp experiments as well (Fig. 5A; mouse: 6.76 ± 1.66 Hz; n = 9 cells; bovine: n = 10 cells RGCs 9.84 ± 2.58). Comparison of the peak firing rates to a full-field light stimulation (Fig. 4B; one second; 550 nm; 5 × 10^14^ photons/cm^2^/s) did not differ significantly between the two species (*P* = 0.1329; mouse = 74.69 ± 41.71 Hz; bovine = 65.44 ± 39.10 Hz). We also compared the onsets of the light-responses between mouse and bovine RGCs to the same light-stimulation (Fig. 4C) and did not observe any temporal differences neither between cells that started responding during light stimulation (*P* = 0.58), nor after it (*P* = 0.19). Comparison of the time to maximal responses (time-to-peak; Fig. 4D), did not yield any significant differences between cells with responses peaking during the light stimulation (*P* = 0.61); however, responses that peaked after the light stimulation exhibited significantly faster kinetics in bovine retina in comparison to the retina of a mouse (*P* = 0.022). Comparing the distributions of the RGC response types in murine and bovine retinas to full-field light flash (Fig. 4E) highlighted the prevalence of ON-responses in the murine retina (ON = 90.6%; OFF = 9.4%) in comparison to a more balanced distribution of responses in the bovine retina (ON = 47.4%; OFF = 52.6%). Comparing basal firing rates of the ON and OFF subtypes did not reveal any significant differences (Fig. 4F). The bovine retina was also sensitive to standardly available retinal pharmacology (Fig. 4G).

The comparison of the RGC-derived light responses with MEAs was complemented with the exploration of intrinsic electrophysiological properties of individual alpha RGCs (mouse: n = 9; bovine: n = 10) using the current-clamp technique in the whole-cell configuration. All alpha RGCs were pooled together because we were unable to identify the specific subgroup of alpha RGCs by using a principal component analysis. Individual data from each RGCs for all the tested parameters (Figs. 5B–K) are available in the Table, together with the averaged data.

Alpha RGCs of mice exhibited significantly more depolarized resting membrane potential (Vm) in comparison to bovine retinal explants (*P* = 0.029; Fig. 5B), which might indicate a difference in composition of ion channels^51^ between these species. As expected, the larger bovine RGCs exhibited significantly smaller input resistance (Rn; Fig. 5C; *P* = 0.001). No significant difference was observed between the time constants of the two species (Fig. 5D; *P* = 0.355). Both mouse and bovine alpha RGCs exhibited similar anomalous rectification in response to hyperpolarization, also called sag (Fig. 5E; *P* = 0.1736) indicating the presence of a hyperpolarization-activated current (*I_h_*).

All RGCs recorded during this study responded to sustained depolarizing current injections with repetitive spiking during the whole duration of the stimulation (Fig. 5F). Similar results have been reported from recordings of rat^52^ and cat^53^ alpha RGCs. The steady (Fig. 5G; *P* = 0.414) and maximal (Fig. 5H; *P* = 0.712) spike frequency did not differ significantly between the two species, showing that both bovine and mouse alpha RGCs could sustain high firing rates for the duration of the stimulation. These regular spiking RGCs clearly displayed decrease in spike frequency over time, particularly during high current injection (Fig. 5F). We calculated the frequency adaptation index to quantify this decrease in spike frequency and we did not observe any significant differences between the two (Fig. 5I; *P* = 0.11). Action potentials of both bovine and murine RGCs exhibited similar amplitudes (Fig. 5J; *P* = 0.19), however those of bovine were on average significantly shorter than those of mice (Fig. 5K; *P* = 0.019).

## Discussion

One of the shortcomings of the dark-adapted ex vivo retina, is the lack of the RPE and therefore disruption of the visual cycle. We show that the choroid-attached ex vivo bovine retina is a readily available model system for retinal physiology with an intact visual cycle and in vivo–like light responses and light adaptation. We use this model to record natural light-evoked responses from individual retinal neurons at photopic light intensities for the very first time in an ex vivo system. To demonstrate the versatility of this model, we record light-evoked mERGs and extracellular RGC responses on a MEA system and also show that choroid-attached retinas are thin enough to allow targeted electrophysiology of individual retinal neurons using standard far-red DIC optics. We demonstrate that choroid-attached retinas adapt to changes in ambient light intensity and that they cannot be bleached by light.

Surprisingly, the A-wave amplitude in isolated retinas, without the RPE, also showed a recovery during the five-minute dark adaptation period (Fig. 2C; *P* < 0.001), albeit to a much smaller extent. It seems likely that this residual component of adaptation reflects light intensity adaptations within the photoreceptor cells that are independent of the bleached state of the opsin. This may involve, for example, arrestin, transducin, and recoverin translocation. In the dark, arrestin leaves the outer segment while transducin and recoverin enter the outer segment.^54^^,^^55^ The net effect is to enhance the gain of the light response. Alternatively, mechanical removal of the choroid may leave residues of RPE on the neural retina, which could recover a fraction of the A-wave amplitude.

To date, the isolated dark-adapted murine retina was the only option to study how retinal neurons encode light stimuli. Attempts to regain light responsiveness in isolated bleached retinas by chemical supplementation has been ineffective. The only commercially available light-activatable chromophore, 9-*cis*-retinal, does recover light sensitivity and blocks the ion channels on the photoreceptor cells that transduce the light signal into an electrical signal.^56^^,^^57^

Although its natural in vivo–like physiology is the fundamental advance of this choroid-attached model, it is also particularly accessible to researchers. It requires less infrastructure compared to experiments on dark-adapted tissue, the bovine retina is cheap and widely available, large eyes are straightforward to dissect and provide ample retinal tissue (Fig. 1) and there are no ethical concerns related to the use of animals solely for research purposes. Unlike humans and mice, however, cattle have a tapetum lucidum,^26^ albeit only in the ventral retina. It is also important to note that, in contrast to mice, cattle possess a visual streak.^25^ The topological location of used explant is therefore an important consideration in further studies. Also, cattle have no transgenic models in comparison to mice.^9^ Numerous studies explored the use of various, commonly available, retinal antibodies in the bovine retina,^33^^–^^37^ which is something we were able to observe as well, along with staining of murine and human cryosections (Supplementary Fig. S4).

However, focusing solely on the ex vivo retina, we observed only few functional differences between murine and bovine retinas (Figs. 4, 5; Table). We find and “extra” brisk ON-OFF alpha cell in the bovine retina (Fig. 3). The retina of cattle seems to exhibit significantly increased basal activity, which may hint to different composition of ion channels,^51^ such as the Kv3 family.^58^ Margolis and Detwiler also proposed^19^ that the basal firing rates may differ between ON and OFF RGCs. Indeed, we observed differences in the distribution of ON and OFF subtypes of RGCs between mouse and bovine, based on onsets of responses to full-field light flash (Fig. 4E). The murine retina was dominated by ON-type RGCs, whereas the bovine retina retained more balance between ON and OFF-type RGCs. However, comparison of the basal firing rates between ON and OFF subtypes did not show any significant differences in either of the species (Fig. 4F). Interestingly, similar balanced distribution of the response types was also observed in human RGCs.^59^ Importantly the distribution of photoreceptors differs vastly between mouse and bovine retinas. In the mouse retina the rod/cone ratio is 30:1,^60^ whereas it was reported that some areas of the bovine retina exhibit as low rod/cone ratio as 3:1.^61^ Such differences may contribute to the difference of synaptic transmissions, hence, affecting the firing rates at the level of RGCs. Our patch-clamp results show that alpha RGCs intrinsic physiological properties are highly conserved between mice and cattle. Alpha cells from other mammalian species, such as rats and cats, share similar properties.^52^^,^^53^^,^^62^ This suggest that these properties might be important for fundamental visual information processing and further validate the use of bovine tissue. Only few species-specific differences were observed such as the resting Vm and the action potential half-width. In mice, three to four different subtypes of alpha RGCs were identified from their morphological and physiological properties.^48^ Our patch-clamp analysis of the intrinsic properties of bovine alpha RGCs shows evidence that similar functional studies can be performed in the bovine retina.

It seems likely that most larger eyes with a relatively small curvature will permit similar choroid-attached preparations. Indeed, one previous report^63^ demonstrated superior long-term survival of photoreceptor cells in ex vivo cultures of choroid-attached porcine retinas compared to isolated porcine retinas. Although this previous work did not demonstrate a closed visual cycle, it confirms the adaptability of this approach and even infers that light sensitivity might be retained for extended periods in culture. We tested the possibility to transduce the cultured bovine choroid-attached ex vivo retinal explants with the recently published 770En_454P(hGRM6)-mCitrine virus,^64^ targeting specifically the ON-bipolar cells in human and murine retinas. Our preliminary results indicate that this is the case for bovine retina as well, with retention of strong ON-bipolar cell specificity (Supplementary Fig. S5).

Ultimately, choroid-attached preparations might be transferable to donated human eyes. Data collected from a human model with a natural response to light will have an unmatched relevance in preclinical research.

## Supplementary Material

Supplement 1

Supplement 2

Supplement 3

Supplement 4

Supplement 5

Supplement 6

Supplement 7

Supplement 8
