# Supplementary material for: The Bovine Ex Vivo Retina: A Versatile Model for Retinal Neuroscience
Source: Invest Ophthalmol Vis Sci. 2023 Aug 23;64(11):29. doi: 10.1167/iovs.64.11.29 (PMC10461644; doi:10.1167/iovs.64.11.29)
Supplement: Supplement 2 [file iovs-64-11-29_s002.pdf]

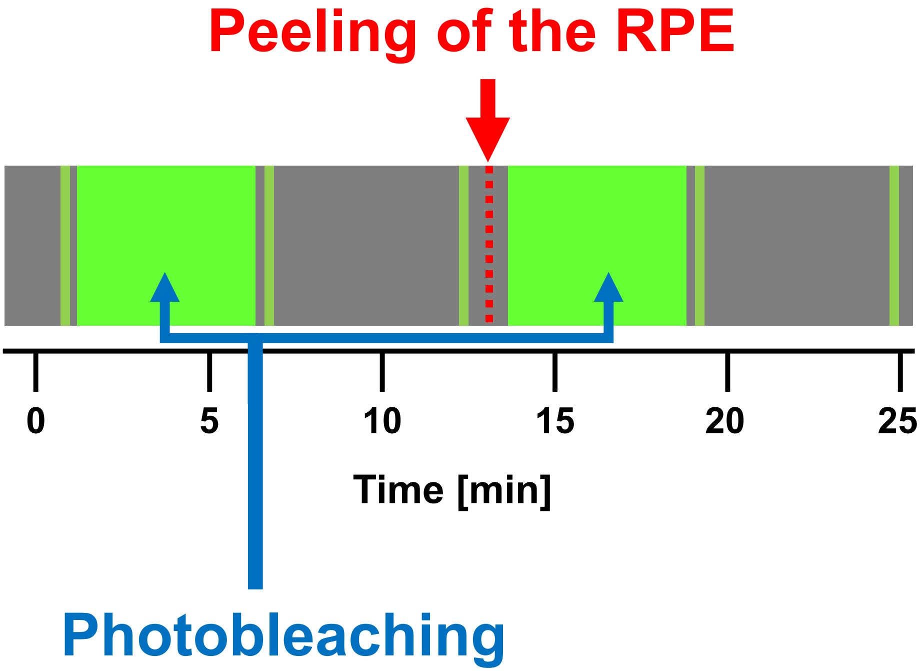

Figure S2. Photobleaching protocol used for MEA recordings: Dark-adapted retinas were exposed to a full-field light flash (550nm; 1s; 3.2 x 10^{15} photons/cm^2/s), followed by 5min of photobleaching (550nm, 5.9 x 10^{15} photons/cm^2/s, 5min). 15s after the photobleaching, another light flash was presented, followed by 5min dark recovery and another light flash. Subsequently, in the scope of ca. 60s, the choroid/RPE was manually peeled off the retina using forceps. As soon as the RPE was peeled, we again photobleached (5min) and presented another light flash (15s after bleaching). This was followed by another period of dark recovery (5min) and a final light flash.
