# Supplementary material for: The Bovine Ex Vivo Retina: A Versatile Model for Retinal Neuroscience
Source: Invest Ophthalmol Vis Sci. 2023 Aug 23;64(11):29. doi: 10.1167/iovs.64.11.29 (PMC10461644; doi:10.1167/iovs.64.11.29)
Supplement: Supplement 3 [file iovs-64-11-29_s003.pdf]

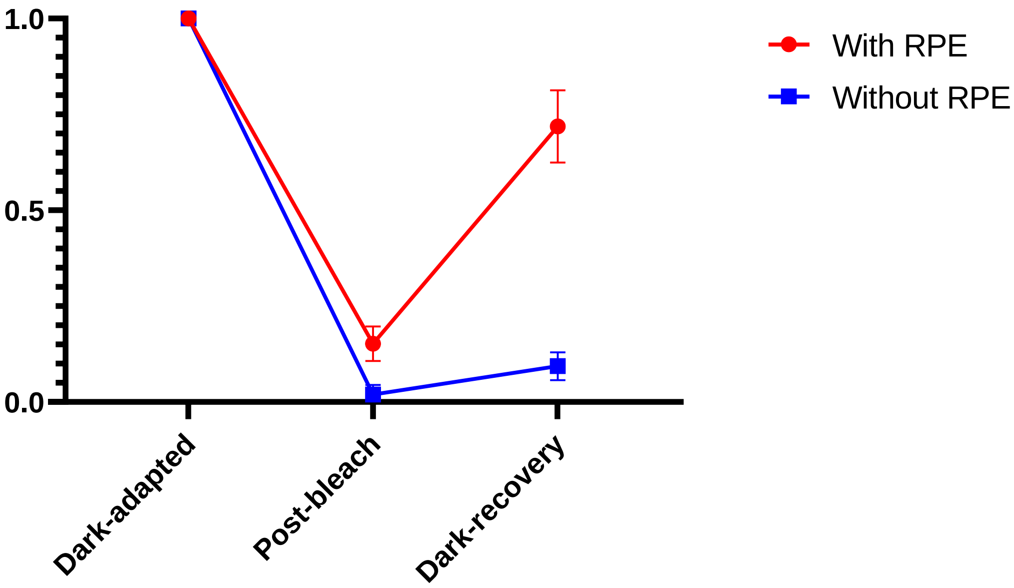

Figure S3: Comparison of normalized A-wave amplitudes in retinal preparations with or without RPE following photobleaching and dark-recovery. Used protocol followed the first 3 light stimulations as depicted in Figure S2. The aim of this experiment was to compare A-wave negative deflections in intact retinas retaining choroid (red trace) and retinas that were isolated from the choroid prior to the start of the recording (blue treace). Dark adapted retinas were exposed to a 1s light flash, that resulted in the mERGs. A-waves derived negative deflections were used as references for normalization across individual electrodes. The intact preparations with RPE (red trace; n=58 electrodes) showed more than 70% recovery to the original amplitudes (71.86±9.43%) in comparison to the preparations without choroid (blue trace; n=59 electrodes) that recovered only to 9% of their original amplitudes (9.34±3.63%).
