# Supplementary material for: The Bovine Ex Vivo Retina: A Versatile Model for Retinal Neuroscience
Source: Invest Ophthalmol Vis Sci. 2023 Aug 23;64(11):29. doi: 10.1167/iovs.64.11.29 (PMC10461644; doi:10.1167/iovs.64.11.29)
Supplement: Supplement 4 [file iovs-64-11-29_s004.pdf]

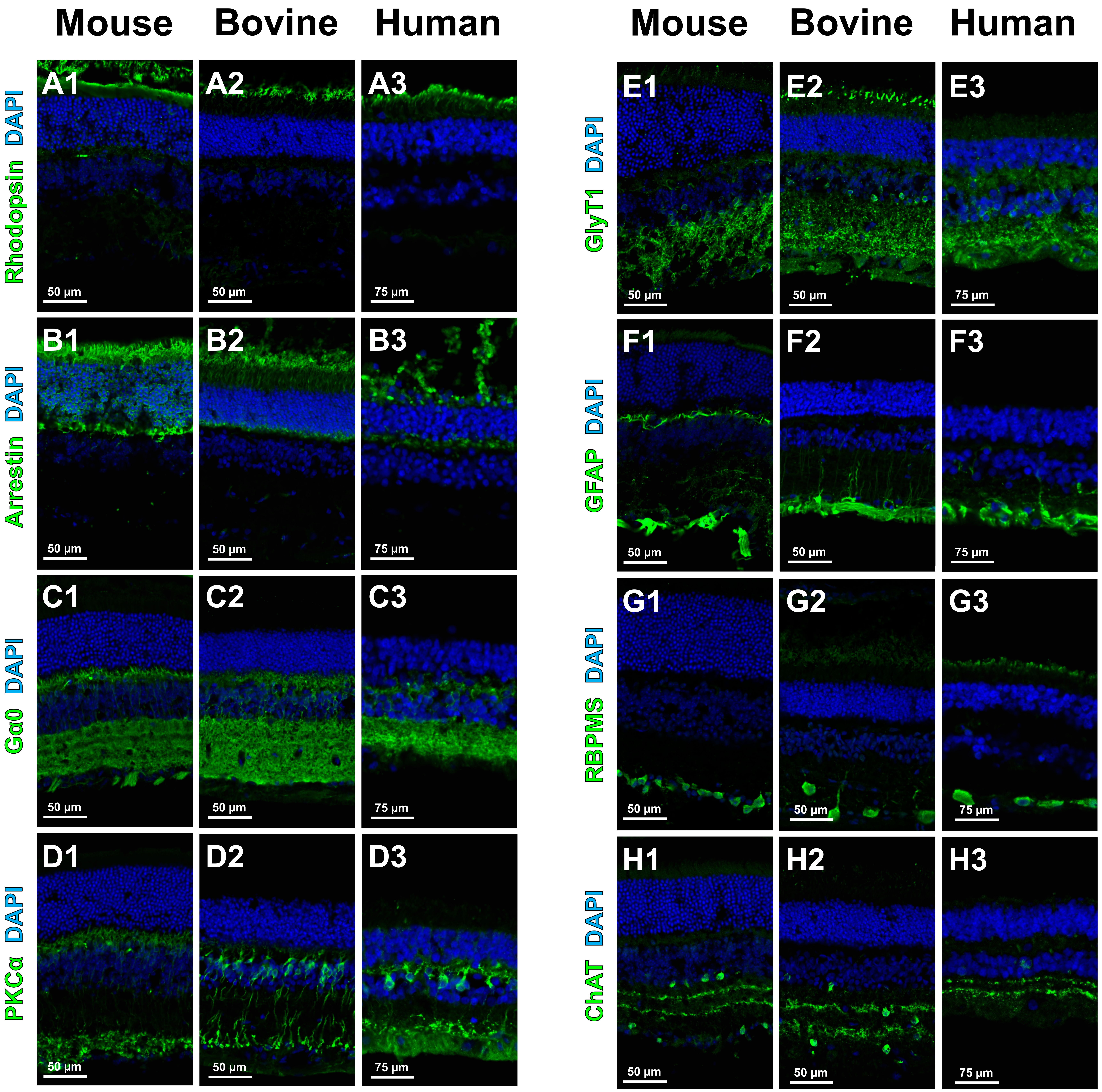

Figure S4. Immunohistochemistry of mouse, bovine, and human cryosections: Blue colour depicts DAPI labelling of the cell nuclei, whereas green indicates antibodies against rhodopsin (**A1-3**), arrestin (**B1-3**), Gα0 (**C1-3**), PKCα (**D1-3**), GlyT1 (**E1-3**), GFAP (**F1-3**), RBPMS (**G1-3**), and ChAT (**H1-3**). Images were taken as single optical sections on a Zeiss LSM880 confocal microscope (20x).
