# Supplementary material for: The Bovine Ex Vivo Retina: A Versatile Model for Retinal Neuroscience
Source: Invest Ophthalmol Vis Sci. 2023 Aug 23;64(11):29. doi: 10.1167/iovs.64.11.29 (PMC10461644; doi:10.1167/iovs.64.11.29)
Supplement: Supplement 5 [file iovs-64-11-29_s005.pdf]

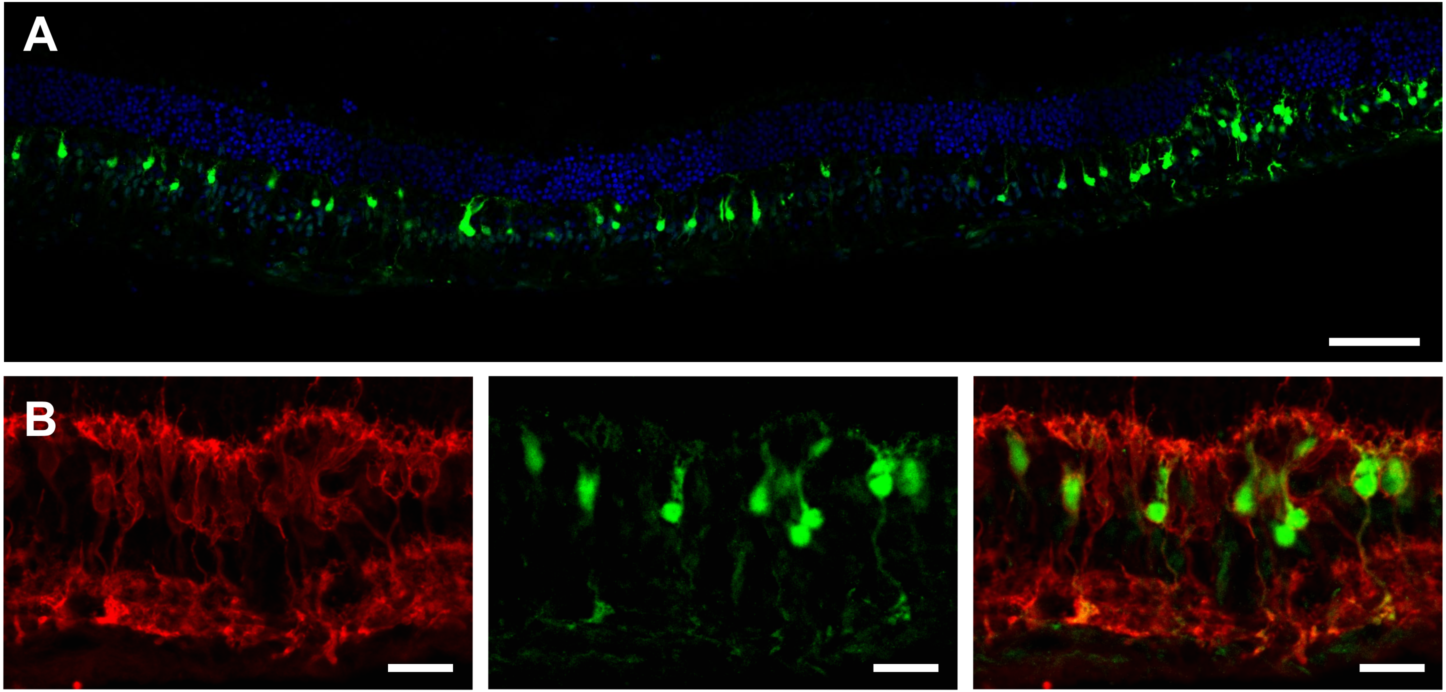

Figure S5. Promoter 770En_454(hGRM6) effectively transduces OBCs of the bovine retina: (**A**) Labeling against the 770En_454P(hGRM6)-mCitrine (green) shows robust transduction. Nuclear DAPI stain in blue, scale bar = 100µm. (**B**) Double labeling with the OBC marker Gα0 (red) and mCitrine (green), shows an OBC-specific preference. Scale bar = 50µm.
