# Supplementary material for: The Bovine Ex Vivo Retina: A Versatile Model for Retinal Neuroscience
Source: Invest Ophthalmol Vis Sci. 2023 Aug 23;64(11):29. doi: 10.1167/iovs.64.11.29 (PMC10461644; doi:10.1167/iovs.64.11.29)
Supplement: Supplement 6 [file iovs-64-11-29_s006.pdf]

| | **Dark-adapted (with choroid)** | **Post-bleach (with choroid)** | **Recovered (with choroid)** | **Post-bleach (without choroid)** | **Recovered (without choroid)** |
|---|---|---|---|---|---|
| **Dark-adapted (with choroid)** | / | <0.001 | <0.001 | <0.001 | <0.001 |
| **Post-bleach (with choroid)** | <0.001 | / | <0.001 | 0.13 | <0.001 |
| **Recovered (with choroid)** | <0.001 | <0.001 | / | <0.001 | <0.001 |
| **Post-bleach (without choroid)** | <0.001 | 0.13 | <0.001 | / | <0.001 |
| **Recovered (without choroid)** | <0.001 | <0.001 | <0.001 | <0.001 | / |

**Table S1. Results of statistical analysis from panel 1E:** The table shows P values from Friedman multiple comparison test of 401 electrodes (n= 9 bovine retinal explants).
