# Supplementary material for: The Bovine Ex Vivo Retina: A Versatile Model for Retinal Neuroscience
Source: Invest Ophthalmol Vis Sci. 2023 Aug 23;64(11):29. doi: 10.1167/iovs.64.11.29 (PMC10461644; doi:10.1167/iovs.64.11.29)
Supplement: Supplement 7 [file iovs-64-11-29_s007.pdf]

	Panel:	Antibody:	Host:	Company:	Catalogue Nr.:	Dilution:
**AB1**	**A**	anti-Rhodopsin 1D4	Mouse	Abcam	ab5417	1:500
	**B**	anti-Arrestin	Goat	Santa Cruz	sc34547	1:250
	**C**	anti-Gα0	Mouse	Millipore	MAB3073	1:750
	**D**	anti-PKCα	Mouse	Santa Cruz	Sc8393	1:750
	**E**	anti-Glyt1	Rabbit	antibodies-online.com	ABIN1841935	1:200
	**F**	anti-GFAP	Rabbit	DAKO	Z0334	1:500
	**G**	anti-RBPMS	Rabbit	MerckMillipore	ABN1362	1:1000
	**H**	anti-choline acetyltransferase	Goat	Millipore	AB144P	1:100
**AB2**	**A,C,D**	anti-Rabbit Alexa Fluor 488	Goat	Invitrogen	A-11008	1:400
	**E,F,G**	anti-Mouse Cyanine 3	Goat	Invitrogen	A-10521	1:400
	**B, H**	anti-Goat Cyanine 3	Donkey	Millipore	APC180C	1:400

Table S2. Antibodies and their concentrations used in Figure S5.
