# Supplementary material for: The Bovine Ex Vivo Retina: A Versatile Model for Retinal Neuroscience
Source: Invest Ophthalmol Vis Sci. 2023 Aug 23;64(11):29. doi: 10.1167/iovs.64.11.29 (PMC10461644; doi:10.1167/iovs.64.11.29)
Supplement: Supplement 8 [file iovs-64-11-29_s008.pdf]

Supplementary materials and methods:

Mouse retinal tissue

The experiments were performed on C57BL/6 wild-type mice. Animal experiments and procedures were in accordance with the Swiss Federal Animal Protection Act and approved by the animal research committee of Bern (approval number BE99/19). Animal care and use conformed to the ARVO Statement for the Use of Animals in Ophthalmic and Vision Research. Animals were maintained under a standard 12 h light-dark cycle. Handling of the tissue for electrophysiological recordings was identical to the procedures used for the bovine retinal tissue.

Human retinal tissue

All procedures were in accord with the tenets of the Declaration of Helsinki and complied with governmental regulations. No ethics approval was required for this type of experiments as per national laws and regulations [Federal Act on Research involving Human Beings (Human Research Act, HRA 810.30, Art. 38)]. The anonymized donor tissue was provided by the Department of Ophthalmology, Inselspital, Bern University Hospital, Bern, Switzerland. We prepared and handled human retinal explants (HREs) as previously described[1,2] and fixed the retinal tissue immediately after the dissection.

Immunohistochemistry staining protocol

Protocols for immunohistochemistry were based on previous publications of the lab[1,3]. Enucleated murine eyes were first prefixed in 4% paraformaldehyde (PFA) in 0.1 M phosphate buffer (pH 7.4) for 60 minutes, followed by dissection (removal of cornea and lens) and fixation for another 30 minutes. Isolated human and bovine retinas were only fixed for 30 minutes. Following fixation, the tissue was cryoprotected in sucrose gradient (10%, 20%, 30% sucrose in PBS) for a minimum of 2 hours in each concentration and overnight for 30% concentration. The tissue was placed in cryomolds filled with Tissue-Tek O.C.T. Compound (Sakura Finetek) and rapidly frozen using liquid-nitrogen-cooled 2-methylbutane. Blocks of frozen tissue were cut using cryostat (Leica) into 14-micron thick sections and collected on SuperFrost Ultra Plus Adhesion slides (Thermo Scientific). 2xNGS (Sigma-Aldrich, G9023) or 2xNDS (Sigma-Aldrich, D966) was used as a blocking solution, composed of 2% bovine serum albumin (BSA; Sigma-Aldrich), 0.3% Triton X-100 (Sigma-Aldrich) and 6% normal serum in TBS. Antibody (AB) solutions were prepared as 1x blocking solution mixed 1:1 with 1xTBS, with addition of respective antibodies (Table S1). Sections were incubated overnight at 4 °C in primary AB solution and 2 h in secondary AB solution with DAPI (0.65 µg/mL, Sigma-Aldrich) at room temperature. Fluoromount mounting medium (Sigma-Aldrich, F4680) was added on the slides and coverslip was carefully placed on top, with the edges being sealed with a clear nail polish. Micrographs were taken on a Zeiss confocal laser scanning microscope (Zeiss LSM 880). The processing of images was done using ImageJ (Rasband WS, version: 1.53m) or ZEISS ZEN lite (ZEISS, version: 3.2).

Hematoxylin-eosin staining protocol

We fixed the choroid-attached retinal flat mounts for 30 min in 4%PFA, and subsequently followed the same cryoprotecting, freezing and cutting protocols as described in the part: Immunohistochemistry staining protocol. To perform the hematoxylin-eosin (H&E) staining we used a H&E Fast Staining Kit (Carl Roth, Art. No. 9194) and followed the instruction manual. To take pictures of the H&E stained sections, we adopted a protocol by Weber & Menko[4] using the blue, green and red filter cubes of the fluorescent microscope equipped with monochrome camera (Zeiss X-cite XYLIS) in order to create composite RGB pictures via ZEISS ZEN lite (ZEISS, version: 3.2).

Bovine retinal cultures and Viral Transduction

The bovine choroid-attached retinas were dissected as previously described (Fig. 2) and placed to Ames' medium containing gentamicin. These pieces were then transported to tissue culturing wells and placed with GC facing upwards. Ames' medium was removed from the wells and warmed and oxygenated culturing medium was added underneath the wells (approximately 1 ml). The composition of the culturing medium was based on a previous study and consisted of DMEM/F12 medium without L-Glutamine (BioConcept, 1-26-F08-I) supplemented with 0.1% BSA, 0.2μM DL-Tocopherol, 1mM fumaric acid, 0.5mM galactose, 1mM glucose, 0.5mM glycine, 10mM HEPES, 0.02μM hydrocortisone, 1μM insulin, L-alanyl-L-Glutamine (25μg/ml), 50μM Mannose, 10μM O-acetyl-L-carnitine hydrochloride, 0.02μM progesterone, 0.1mM putrescine dihydrochloride, 0.35μM retinol, 0.3μM retinyl acetate, 13mM sodium bicarbonate, 0.05μM sodium selenite, 0.003μM 3,3',5-Triiodo-L-thyronine sodium salt, 3mM taurine, 0.5mM ascorbic acid. All components were supplied by Sigma-Aldrich. The medium was additionally supplemented with gentamicin and penicillin-streptomycin. The explants were cultured for at least 2 hours, before addition of 10 μl of viral solution (scAAV2(7m8)-770En_454P(hGRM6)-mCitrine) on top of the explants as described elsewhere[1]. From that point on, the medium exchange occurred every 48 hours, where 500 μl of medium was discarded and replaced by 510 μl of fresh warm medium. The explants were cultured at 37°C under 95% Air / 5% CO_2 atmosphere for 20 days before fixed and processed for immunohistochemistry.

MEA light-response analysis

Following spike sorting using Offline Sorter (Plexon), the time points of spike occurrences were extracted in respect to light stimulations and time binned (bin width: 0.1s). Cells were defined as light responsive using two parameters, with the requirement of fulfilling both: (i) Threshold (TR) defined as a change in firing rate (baseline + 3*SD), or at least 40 Hz in case of cells with no basal activity, between the average frequency prior to light stimulation and at least 1 time bin during or after the light flash and (ii) using light response index (LRI)[3]. Only cells with the respective LRI > 0.2 and TR crossing were considered light responsive. We focused on 4 parameters – baseline firing rate (1s before light stimulation), peak firing rate, onset of light response (defined as first occurrence of TR crossing) and time of maximal response (time bin during or after light stimulation with highest firing rate).

MEA pharmacology protocols

The photoreceptor-derived light responses were blocked with retinal pharmacology as described elsewhere[5,6]. In short addition of the mGluR6 agonist L-(+)-2-amino-4-phosphonobutyric acid (L-AP4; Tocris; 20 μM; 10min) abolished the ON-components of the light responses, whereas addition of AMPA/kainite receptor antagonist 6-cyano-7-nitroquinoxaline-2,3-dione (CNQX; Tocris ; 20 μM; 10min) and the NMDA antagonist DL-(-)-2-Amino-5-phosphonopentanoic acid (DL-APV; Tocris; 40 μM; 10min) resulted in complete blockade of the upstream input to the RGCs.

Analysis of passive and active alpha RGC membrane properties using single-cell patch clamp recordings

The resting membrane potential was obtained with the I-Clamp to zero current mode. We calculated the input resistance (Rn) from the change in Vm produced by small-amplitude hyperpolarizing current injections (ΔVm ~ 5 mV). Small hyperpolarizing current (ΔVm ~ 5 mV) were also used to calculate the membrane time constant. We used 1-s long hyperpolarizing current steps to measure the sag (gradual decay in the membrane hyperpolarization evoked by negative current injection). We determined the amount of rectification by measuring the positive shift in Vm back toward the resting level when the peak hyperpolarization reached approximately −95 mV during 1s pulses of negative current. Finally, three levels of 1-s long depolarizing current steps were injected to the cell, one close to the threshold, one inducing the highest firing rate and a current in between (midrange current). The action potential amplitude and spike widths were measured on spikes evoked by the midrange current steps to reduce the effect of high spiking frequency on spike width. The steady frequency, maximal frequency, and the frequency adaptation (FA) index were calculated from the largest depolarizing current step. The steady frequency is obtained from the

last 3 action potentials of the 1s depolarizing current injection and the maximal frequency from the first 3 action potentials. The frequency adaptation index is calculated as follow:

FA= (maximal frequency - steady frequency) / maximal frequency

The resulting index ranges from 0 (no change in firing rate) to 1 (decreasing firing rate).

Supplementary references:

1. Hulliger, E. C., Hostettler, S. M. & Kleinlogel, S. Empowering Retinal Gene Therapy with a Specific Promoter for Human Rod and Cone ON-Bipolar Cells. *Mol. Ther. - Methods Clin. Dev.* **17**, 505–519 (2020).
2. van Wyk, M., Hulliger, E. C., Girod, L., Ebneter, A. & Kleinlogel, S. Present molecular limitations of ON-bipolar cell targeted gene therapy. *Front. Neurosci.* **11**, (2017).
3. Kralik, J., Van Wyk, M., Stocker, N. & Kleinlogel, S. Bipolar Cell Targeted Optogenetic Gene Therapy Restores Parallel Retinal Signaling And High-Level Vision In The Degenerated Retina. *Commun Biol* **5**, (2022).
4. Weber, G. F. & Menko, A. S. Color image acquisition using a monochrome camera and standard fluorescence filter cubes. *Biotechniques* **38**, 52–56 (2005).
5. Kralik, J. & Kleinlogel, S. Functional Availability of ON-Bipolar Cells in the Degenerated Retina: Timing and Longevity of an Optogenetic Gene Therapy. *Int. J. Mol. Sci. 2021, Vol. 22, Page 11515* **22**, 11515 (2021).
6. van Wyk, M., Pielecka-Fortuna, J., Löwel, S. & Kleinlogel, S. Restoring the ON Switch in Blind Retinas: Opto-mGluR6, a Next-Generation, Cell-Tailored Optogenetic Tool. *PLoS Biol.* **13**, (2015).
