# Supplementary material for: The Bovine Ex Vivo Retina: A Versatile Model for Retinal Neuroscience
Source: Invest Ophthalmol Vis Sci. 2023 Aug 23;64(11):29. doi: 10.1167/iovs.64.11.29 (PMC10461644; doi:10.1167/iovs.64.11.29)
Supplement: Supplement 1 [file iovs-64-11-29_s001.pdf]

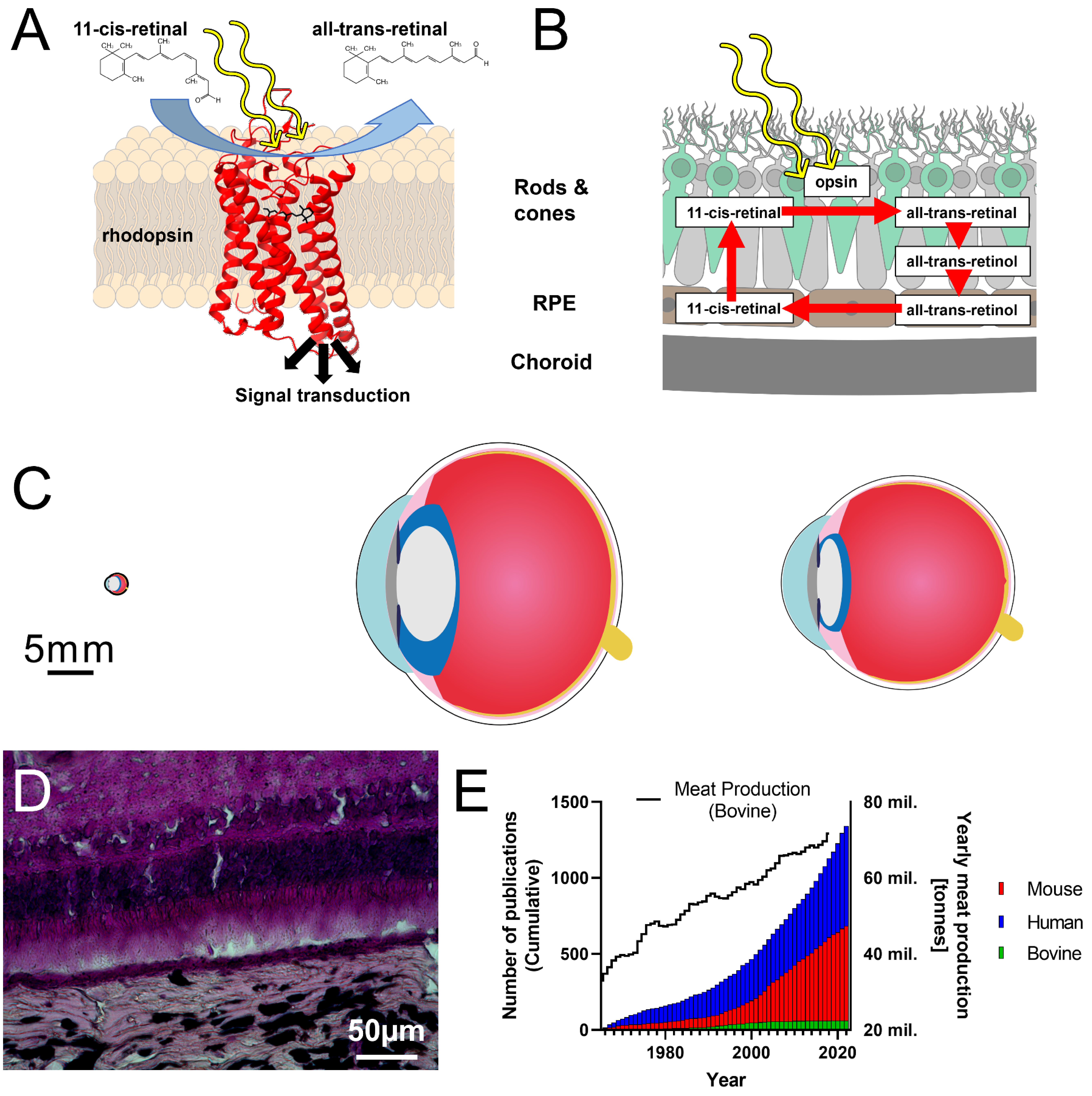

Figure S1: The RPE forms an integral part of the visual cycle. (**A**) The light-sensing GPCRs in the photoreceptors (rhodopsin and cone opsin) only respond to light when bound to the 11-*cis*-retinal chromophore. Absorbing a photon (yellow arrow) photoisomerizes 11-*cis*-retinal to all-*trans*-retinal, that is released by the opsin. (**B**) Released all-trans retinal is converted back to 11-*cis*-retinal in series of chemical reactions within the RPE and transported back to the opsin to restore light sensitivity. (**C**) Relative comparison of the size of murine (left), bovine (middle) and human (right) eye. (**D**) H&E staining of choroid-attached retinal *ex-vivo* explant (magnification= 20x). (**E**) Cumulative number of publications between years 1965 and 2022 focusing on retinal electrophysiology of mouse (red), bovine (green) and human (blue). Left Y-axis corresponds to these datasets. Data obtained via PubMed search engine (24.11.2022), using the formula ((electrophysiology) AND (retina)), combined with ((mouse) OR (murine)); ((bovine) OR (cattle) OR (cow)); (human). The black line displays production of the bovine meat industry worldwide between the years 1965 and 2018[29], corresponds to the right Y-axis.
